# Feasibility of a dose-intensive CMF regimen with granulocyte colony-stimulating factor as adjuvant therapy in premenopausal patients with node-positive breast cancer

**DOI:** 10.1054/bjoc.2000.1242

**Published:** 2000-05-18

**Authors:** A M E Bos, H de Graaf, E G E de Vries, H Piersma, P H B Willemse

**Affiliations:** Division of Medical Oncology, Department of Internal Medicine, University Hospital, PO Box 30 001, Groningen, RB, 9700, The Netherlands; Department of Internal Medicine, Medical Center Leeuwarden, Leeuwarden, The Netherlands; Department of Internal Medicine, Martini Hospital, Groningen, The Netherlands

**Keywords:** adjuvant chemotherapy, breast cancer, CMF, dose intensity, granulocyte colony stimulating factor, premenopausal

## Abstract

Our aim was to study the feasibility of an intensified intravenous CMF (cyclophosphamide, methotrexate and 5-fluorouracil) schedule with the aim to escalate dose intensity (DI). Twenty-three premenopausal breast cancer patients received 6 cycles of adjuvant CMF intravenously on days 1 and 8 every 3 weeks and granulocyte colony-stimulating factor days 9–18. Endpoints were DI and toxicity. Twenty-one out of 23 patients (91%) received the projected total dose and reached ≥ 85% of the projected DI. Compared to ‘classical’ CMF, all patients reached ≥ 111% DI. Nine patients received the planned schedule without delay. Thirteen patients (57%) were treated for infection and four patients (17%) were hospitalized for febrile neutropenia. Twelve patients received red blood cell transfusions (52%). Radiation therapy (*n*= 6) had no adverse impact on dose intensity or haematological toxicity. This dose-intensified CMF schedule was accompanied by enhanced haematological toxicity with clinical sequelae, namely fever, intravenous antibiotics and red blood cell transfusions, but allows a high dose intensity in a majority of patients. © 2000 Cancer Research Campaign


British Journal of Cancer (2000) **82**(12), 1920–1924
© 2000 Cancer Research Campaign
doi: 10.1054/ bjoc.2000.1242, available online at http://www.idealibrary.com on IDEAL®

### Feasibility of a dose-intensive CMF regimen with granulocyte colony-stimulating factor as adjuvant therapy in premenopausal patients with node-positive breast cancer

**AME Bos[1], H de Graaf[2], EGE de Vries[1], H Piersma[3] and PHB Willemse[1]**

[1]Division of Medical Oncology, Department of Internal Medicine, University Hospital, PO Box 30 001, 9700 RB Groningen, The Netherlands; [2]Department of Internal Medicine, Medical Center Leeuwarden, Leeuwarden, The Netherlands; [3]Department of Internal Medicine, Martini Hospital, Groningen, The Netherlands

**Summary** Our aim was to study the feasibility of an intensified intravenous CMF (cyclophosphamide, methotrexate and 5-fluorouracil) schedule with the aim to escalate dose intensity (DI). Twenty-three premenopausal breast cancer patients received 6 cycles of adjuvant CMF intravenously on days 1 and 8 every 3 weeks and granulocyte colony-stimulating factor days 9–18. Endpoints were DI and toxicity. Twenty-one out of 23 patients (91%) received the projected total dose and reached ≥ 85% of the projected DI. Compared to 'classical' CMF, all patients reached ≥ 111% DI. Nine patients received the planned schedule without delay. Thirteen patients (57%) were treated for infection and four patients (17%) were hospitalized for febrile neutropenia. Twelve patients received red blood cell transfusions (52%). Radiation therapy (*n* = 6) had no adverse impact on dose intensity or haematological toxicity. This dose-intensified CMF schedule was accompanied by enhanced haematological toxicity with clinical sequelae, namely fever, intravenous antibiotics and red blood cell transfusions, but allows a high dose intensity in a majority of patients. © 2000 Cancer Research Campaign

**Keywords**: adjuvant chemotherapy; breast cancer; CMF; dose intensity; granulocyte colony stimulating factor; premenopausal

Cyclophosphamide, methotrexate and 5-fluorouracil (CMF) is widely used as a chemotherapy combination for the adjuvant treatment of breast cancer. The 'classical' CMF regimen comprises 6 cycles of oral cyclophosphamide (100 mg $m^{-2}$ $day^{-1}$) days 1–14 with intravenous (i.v.) methotrexate (40 mg $m^{-2}$) and i.v. 5-fluorouracil (600 mg $m^{-2}$) on days 1 and 8, repeated every 28 days (Bonadonna and Valagussa, 1981). To improve the therapeutic index, the dosages, schedule and route of administration of CMF have been widely varied. Several trials have suggested that relapse-free survival and overall survival not only depend on the total dose of the cytotoxic drugs actually administered, but the more so on the dose intensity, i.e. the amount of drug given per unit of time (Bonadonna and Valagussa, 1981; Hryniuk and Bush, 1984; Hryniuk and Levine, 1986; Hryniuk et al, 1987; Tannock et al, 1988; Ang et al, 1989; Engelsman et al, 1991; Wood et al, 1994).

Based on the assumptions that compliance with oral cyclophosphamide would be less than when the drug was given i.v. and that variability in absorption of cyclophosphamide by the oral route could lead to variable bio-availability, several studies have used i.v. CMF schedules. A potential advantage of the i.v. regimens is the easier possibility of a combination with a haematopoietic growth factor such as granulocyte colony-stimulating factor (G-CSF). G-CSF stimulates the recovery of granulocytes after chemotherapy. G-CSF has been used to enhance dose intensity by shortening the interval between cycles or by increase in dosage (Bronchud et al, 1989; Neidhart et al, 1989; Crawford et al, 1991; Lieschke and Burgess, 1992; Biesma et al, 1992; De Graaf et al, 1996; Ribas et al, 1996).

The aim of the present prospective study was to evaluate the feasibility of a regimen with an intensified i.v. CMF schedule supported by G-CSF and administered every 3 weeks, reaching a projected dose intensity (DI) of 143% compared to 'classical' CMF.

*Received 7 April 1999*
*Revised 13 August 1999*
*Accepted 25 January 2000*

*Correspondence to:* AME Bos

### PATIENTS AND METHODS

Eligible were premenopausal women who were considered for adjuvant chemotherapy with CMF. Primary treatment consisted of a modified radical mastectomy or breast-conserving surgery. Patients were ineligible if they had renal impairment (serum creatinine level > 120 µmol $l^{-1}$), abnormal liver function (bilirubin level > 25 mmol $l^{-1}$) or abnormal baseline marrow reserve (leucocyte count < $3.0 \times 10^9$ $l^{-1}$, platelet count < $150 \times 10^9$ $l^{-1}$).

Patients received cyclophosphamide 750 mg $m^{-2}$, methotrexate 40 mg $m^{-2}$ and 5-fluorouracil 600 mg $m^{-2}$, all i.v. on days 1 and 8, repeated every 21 days, for a total of 6 cycles. The administration of the chemotherapy on days 1 and 8 were defined as two separate courses (A and B), so patients received a total of 12 courses. G-CSF (Neupogen, Roche, Mijdrecht, The Netherlands) was administered in a dose of 300 µg subcutaneously once a day on days 9–18 of each cycle. Blood counts were collected on days 1 and 8 before i.v. administration of the chemotherapeutic drugs. The chemotherapy was administered if the leucocyte count was > $2.5 \times 10^9$ $l^{-1}$ on day 1 or > $1.0 \times 10^9$ $l^{-1}$ on day 8 and if the platelet count was > $75 \times 10^9$ $l^{-1}$ on day 1 and > $50 \times 10^9$ $l^{-1}$ on day 8. These non-conventional thresholds, which were allowed by the support of G-CSF, were applied to minimize the delay of treatment due to myelosuppression and to achieve a dose-intensive CMF regimen. In the event of myelosuppression on the planned day of drug administration, treatment was delayed for 1 week. No dose reductions were scheduled for nadir values or intercurrent fever. Red blood cell transfusion was administered for haemoglobin values < 6.5 mmol $l^{-1}$.

Radiation therapy was administered in case of involvement of more than three positive lymph nodes, extranodal tumour growth, multifocal tumour or breast lymphangitis. Radiotherapy was administered concomitantly with CMF chemotherapy.

Toxicity was recorded using the WHO criteria (WHO, 1979).

The total dose of the chemotherapeutic drugs was expressed as the percentage of the actual amount administered divided by the projected amount, in which each drug was given equal value. The DI was given as a percentage of the total dose administered per unit time (weeks), divided by the actual duration of treatment. The study was approved by the Medical Ethical Committee and all patients gave informed consent.

The $\chi^2$ test (Mantel–Haenszel) was used for statistical analysis with the exception of the analysis of the leucocyte counts related to Figure 1. For this purpose Friedman's test (two-way rank analysis) was used together with Duncan's test for correction of multiple comparisons. The confidence intervals were 95%.

### RESULTS

### Patient characteristics

Over a period of 1 year, 23 women entered the study. Twenty-one patients had undergone a modified radical mastectomy and two patients breast-conserving surgery. Twenty-two patients had lymph node involvement, two had more than four positive nodes, one patient was node-negative. Six patients received loco-regional radiation therapy, including two with breast conserving therapy. The median start of the chemotherapy was 19 days (range 14–48) and of radiotherapy 64 days after surgery (range 43–78 days). The patients' characteristics are shown in Table 1.

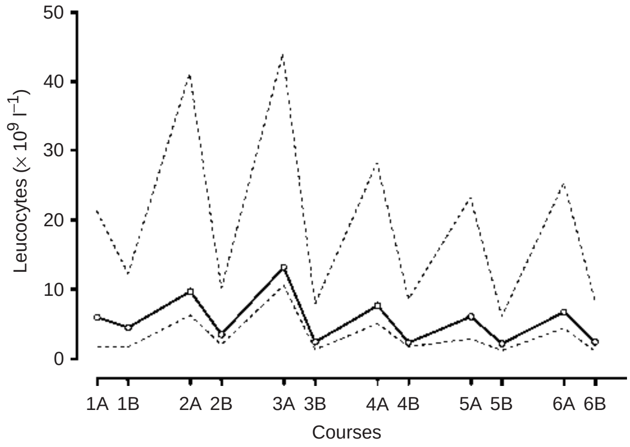

**Figure 1** Leucocyte count (median and range) at the start of each course

**Table 1** Patient characteristics ($n$ = 23)

| | |
|---|---|
| Age (years) | |
| Median | 44 |
| Range | 26–55 |
| Primary tumour | |
| $pT_1$ | 4 |
| $pT_2$ | 19 |
| Axillary lymph nodes examined | |
| Median | 10 |
| Range | 1–16 |
| Axillary lymph nodes involved | |
| Median | 1 |
| Range | 1–8 |
| Surgical treatment | |
| Modified mastectomy | 21 |
| Breast-conserving surgery | 2 |
| Locoregional radiotherapy | 6 |

### Dose intensity

Two patients did not receive all courses of chemotherapy. One patient had fever with leukopenia and skipped course 4B. Another patient did not receive the last course (6B) due to haematological toxicity. A total of 274 out of 276 courses were completed. Table 2 shows the actually achieved DI as a percentage of the projected DI (range 78–100%) and the actually achieved DI compared to 'classical' CMF (range 111–143%). In 21 patients the actually delivered DI was ≥ 85% of the projected DI, which is the equivalent of ≥ 120% compared to 'classical' CMF.

### Delay of treatment

Out of these 23 patients, ten received all treatment as planned; delay of treatment occurred in 13 patients (57%). A total of 17 courses out of 274 (6.2%) were delayed for a median of 1 week (range 1–3 weeks). The total delay was 23 weeks (5.6%) on a projected total treatment duration of 414 weeks for all patients. The reasons for delay of chemotherapy are listed in Table 3. Delay for insufficient marrow recovery and for fever and infection were the most important causes.

### Toxicity

Figure 1 shows the median leucocyte count with ranges at the start of the courses. Over time, the median leucocyte count on day 8 declined, suggesting cumulative toxicity. Moreover, the absolute increase of leucocytes during G-CSF administration (i.e. the reserve-capacity) declined. The relative increase of leucocytes related to the nadir in these cycles, however, were not different.

**Table 2** Actually achieved dose intensity (DI) compared to 'classical' CMF regimen

| Achieved dose intensity (% of projected DI) | Number of patients | Achieved DI compared to 'classical' CMF |
|---|---|---|
| DI 100% | 9 | DI 143% |
| DI 85%–100% | 12 | DI 120–143% |
| DI < 85% | 2 | DI < 120% |
| Median DI: 95% (range 78–100%) | Total: 23 | Median DI: 135% (range 111–143%) |

**Table 3** Reasons for delay of chemotherapy

| | Number of courses (total = 276) | Number of weeks delay[b] |
|---|---|---|
| Criteria for treatment delay: (× $10^9$ $l^{-1}$) | | |
| Course A (day 1): | | |
| Leucocyte count ≤ 2.5 | 3 | 3 |
| Platelet count ≤ 75 | 1 | 1 |
| Both | 1 | 2 |
| Course B (day 8): | | |
| Leucocyte count ≤ 1.0 | 1 | 1 |
| Clinical events: | | |
| Infection grade 2 | 6 | 8 |
| Infection grade 3 | 3 | 4 |
| Nausea/vomiting grade 3 | 1 | 1 |
| Surgery[a] | 1 | 3 |
| Total | 17 (6.2%) | 23 (5.6%) |

[a]Secondary mastectomy after breast-conserving therapy for extensive DCIS (ductal carcinoma in situ). [b]The projected cumulative time on treatment for all patients is 414 treatment-weeks

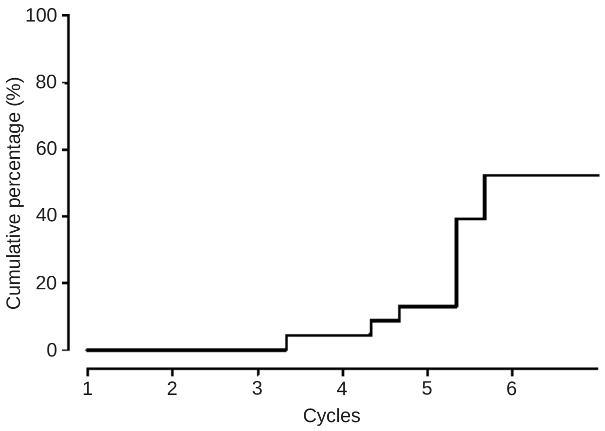

**Figure 3** Time to the first red blood cell (RBC) transfusion

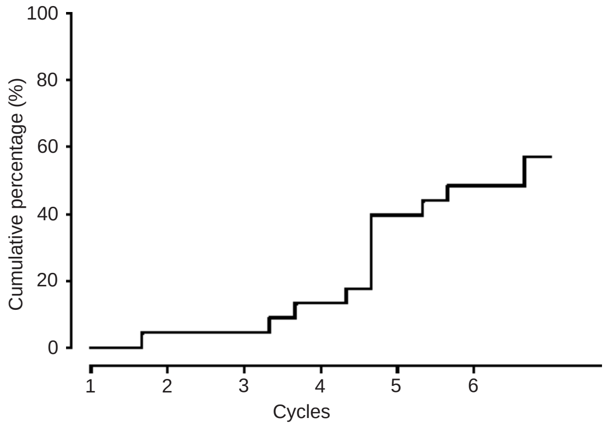

**Figure 2** Time to the first episode of infection treated by antibiotics

Overall, these changes indicate a gradual fall in bone marrow reserve capacity.

Thirteen out of 23 patients (57%) were at least once (range 1–3) affected by fever grade 2 (temperature > 38.0°C) and were treated by oral or i.v. antibiotics. Eleven patients (48%) were treated by oral antibiotics (infection grade 2). Four out of 23 patients (17%) had fever with neutropenia and were admitted for i.v. antibiotics (infection grade 3). The time to the first episode of infection is shown in Figure 2. One prophylactic transfusion of platelets was given for grade IV thrombocytopenia without bleeding. Red blood cell (RBC) transfusions were administered to 12 patients (52%) at a median value of haemoglobin of 5.5 mmol $l^{-1}$. The median number of transfusions was 3 units (range 2–6), for a total of 45 units. Figure 3 shows the cumulative probability of the first RBC transfusion during treatment.

The main toxicity related to the use of G-CSF was mild bone pain in seven patients (mainly in the first two cycles during the last days of G-CSF) and musculoskeletal pain in two patients and was not a reason to withhold its administration.

### Radiotherapy

Six patients received radiation therapy. All six patients received the projected total dose. The actual achieved DI was for three patients 100%, for two patients 95% and for one patient 90%. Insufficient leucocyte recovery (leucocyte count ≤ 3 × $10^9$ $l^{-1}$) occurred in 23 of 72 courses (32%) versus in 64 of 204 courses (32%) without radiotherapy (ns). One patient treated with radiotherapy was hospitalized for infection grade 3 and RBC transfusion was administered to three patients (ns).

### DISCUSSION

We have studied the feasibility of an accelerated CMF schedule aiming to reach a higher dose intensity. The dose-intensification was achieved by shortening the cycle interval and by slightly increasing the dose of cyclophosphamide, supported by G-CSF. The dose intensity for cyclophosphamide was 500 mg $m^{-2}$ $week^{-1}$ i.v., a factor 1.43 compared to the 'classical' CMF regimen (350 mg $m^{-2}$ $week^{-1}$ orally). The dose intensity for methotrexate and 5-fluorouracil was 133% compared to the oral schedule. With this modified schedule, the median actually achieved dose intensity was 135% compared to the 'classical' CMF. Recently, Goldhirsch et al concluded that the many variations in CMF regimens did not improve results (Goldhirsch et al, 1998*a*, 1998*b*). However, several studies have suggested that a higher dose or dose intensity of chemotherapy may improve disease-free and overall survival (Bonadonna and Valagussa, 1981; Hryniuk and Bush, 1984; Hryniuk and Levine, 1986; Hryniuk et al, 1987; Tannock et al, 1988; Ang et al, 1989; Engelsman et al, 1991; Wood et al, 1994). Bonadonna and Valagussa (1981) suggested after a retrospective analysis, that the effectiveness of adjuvant CMF depends on the total dose actually administered. CMF was only useful when given ≥ 85% of the planned dose (Bonadonna and Valagussa, 1981). Wood et al reported the results of a prospective, randomized trial of adjuvant cyclophosphamide, doxorubicin and 5-fluorouracil in three dose levels (Wood et al, 1994). The women treated with a moderate or high dose intensity had a significantly longer disease-free and overall survival than those treated with a low dose intensity. Tannock et al reported a reduction in response rate and overall survival in patients who received the lower (50%)

 *© 2000 Cancer Research Campaign*

dose arm compared to the standard intravenous CMF in metastatic disease (Tannock et al, 1988).

An advantage of giving cyclophosphamide i.v. is the possibility to start G-CSF immediately after the second i.v. dose from day 8 onwards and thus shorten the interval between the cycles. Several studies examining the route of administration have been published (Engelsman et al, 1991; Lindeman et al, 1992). An EORTC randomized study has compared 'classical' CMF with i.v. CMF (cyclophosphamide 600 mg $m^{-2}$, methotrexate 40 mg $m^{-2}$ and 5-fluorouracil 600 mg $m^{-2}$, all i.v. on day 1) in 254 eligible patients with metastatic breast cancer (Engelsman et al, 1991). The response rate after 'classical' CMF was 48% compared with 29% for i.v. CMF, but in the 'classical' CMF a higher dose intensity was achieved.

In the present trial the criteria for delay of courses due to myelosuppression were less restricted than in most adjuvant breast cancer protocols, which was allowed by the support of G-CSF. In the hypothetical case that the criteria would have been chosen more stringent (e.g. leucocyte count > $3.0 \times 10^9$ $l^{-1}$ and platelet count > $100 \times 10^9$ $l^{-1}$ on day 1 and leucocyte count > $2.0 \times 10^9$ $l^{-1}$ and platelet count > $75 \times 10^9$ $l^{-1}$ on day 8), course A would have been postponed 16 instead of 5 weeks (11.6% vs 3.6%) and course B 41 weeks instead of once (29.7% vs 0.7%). This would have resulted in 19 patients (83%) with delay of treatment and an estimated achieved DI of 84% at most. The effect of G-CSF on DI of standard oral adjuvant CMF in 123 patients with breast cancer was studied by De Graaf et al. Without G-CSF the leucocyte count on day one was ≤ $3 \times 10^9$ $l^{-1}$ in 21% of the courses (De Graaf et al, 1996). In our study, the leucocyte count was ≤ $3 \times 10^9$ $l^{-1}$ in 32% of the courses. Besides, 17% of the patients had to be treated with i.v. antibiotics and 52% of the patients needed RBC transfusions. With the 'classical' CMF regimen RBC transfusions are rarely given.

G-CSF was administered on days 9–18 and was not given simultaneously with chemotherapy to avoid enhanced myelotoxicity (ASCO, 1994). Recently, Tjan-Heijnen et al reported that, in small-cell lung carcinoma patients, stopping G-CSF administration 48 h before the next chemotherapy course increased chemotherapy-associated leucopenia and thrombocytopenia, implying a carry-over effect in the next cycles (Tjan-Heijnen et al, 1998). It might be that stopping G-CSF earlier would yield better results.

Several investigators showed that radiotherapy could have a negative effect on marrow recovery and on the dose intensity in combination with chemotherapy (Holland et al, 1980; Cooper et al, 1981; Levine et al, 1984; De Graaf et al, 1996). This was especially seen when G-CSF was administered in conjunction with the radiotherapy, leading to additional delays for thrombocytopenia. However, Pronzato et al did not find a negative effect of radiotherapy on the dose intensity of adjuvant CMF (Pronzato et al, 1993). In our study, the subgroup of patients having received radiation therapy had equal dose intensity and there was no difference in infection rate. We can not therefore in this small group confirm an adverse impact of radiotherapy.

We conclude that this modified i.v. CMF regimen carries enhanced haematological toxicity with clinical sequelae (namely fever, i.v. antibiotics and many RBC transfusions), but allows a high dose intensity in a majority of patients. This dose-intensive CMF schedule could be the basis for a randomized phase III study to compare with 'classical' CMF. Such a study should also examine dose-intensity, toxicity, cost and quality of life. It should be possible to use erythopoietin for treatment of anaemia and prophylactic antibiotics to prevent infections. Also, repeated peripheral stem cell support could be used to achieve high dose intensity with less haematological toxicity.

### ACKNOWLEDGEMENTS

We would like to acknowledge WJ Sluiter (PhD) for his statistical assistance and M Luchies, G Dijkinga and J van der Til, oncology nurses in the Martini Hospital, for their help in patient guidance and support.

### REFERENCES

Ang PT, Buzdar AU, Smith TL, Kau S and Hortobagyi GN (1989) Analysis of dose intensity in doxorubicin-containing adjuvant chemotherapy in stage II and III breast carcinoma. *J Clin Oncol* **7**: 1677–1684

ASCO Ad Hoc Colony-Stimulating Factor Guideline Expert Panel (1994) The American Society of Clinical Oncology recommendations for the use of hematopoietic colony-stimulating factors: evidence-based, clinical practice guidelines. *J Clin Oncol* **12**: 2471–2508

Biesma B, Vellenga E, Willemse PH and De Vries EG (1992) Effects of hematopoietic growth factors on chemotherapy-induced myelosuppression. *Crit Rev Oncol Hematol* **13**: 107–134

Bonadonna G and Valagussa P (1981) Dose–response effect of adjuvant chemotherapy in breast cancer. *N Engl J Med* **304**: 10–15

Bronchud MH, Howell A, Crowther D, Hopwood P, Souza L et al (1989) The use of granulocyte colony-stimulating factor to increase the intensity of treatment with doxorubicin in patients with advanced breast and ovarian cancer. *Br J Cancer* **60**: 121–125

Cooper MR, Rhyne AL, Muss HB, Ferree C, Richards F 2d, White DR, Stuart JJ, Jackson DV, Howard V, Shore A, Spurr CL (1981) A randomized comparative trial of chemotherapy and irradiation therapy for stage II breast cancer. *Cancer* **47**: 2833–2839

Crawford J, Ozer H, Stoller R, Johnson D, Lyman G, Tabbara I, Kris M, Grous J, Picozzi V, Rausch G (1991) Reduction by granulocyte colony-stimulating factor of fever and neutropenia induced by chemotherapy in patients with small-cell lung cancer. *N Engl J Med* **325**: 164–170

De Graaf H, Willemse PH, Bong SB, Piersma H, Tjabbes T, van Veelen H, Coenen JL, de Vries EG (1996) Dose intensity of standard adjuvant CMF with granulocyte colony-stimulating factor for premenopausal patients with node-positive breast cancer. *Oncology* **53**: 289–294

Engelsman E, Klijn JCM, Rubens RD, Wildiers J, Beex LVAM, Nooij MA, Rotmensz N, Sylvester R (1991) 'Classical' CMF versus a 3-weekly intravenous CMF schedule in postmenopausal patients with advanced breast cancer. *Eur J Cancer* **27**: 966–970

Goldhirsch A, Colleoni M, Coates AS, Castiglione-Gertsch M, Gelber RD (1998*a*) Adding adjuvant CMF chemotherapy to either radiotherapy or tamoxifen: are all CMFs alike? *Ann Oncol* **9**: 489–493

Goldhirsch A, Coates AS, Colleoni M, Castiglione-Gertsch M, Gelber RD (1998*b*) Adjuvant chemoendocrine therapy in postmenopausal breast cancer: cyclophosphamide, methotrexate and fluorouracil dose and schedule may make a difference. *J Clin Oncol* **16**: 1358–1362

Holland JF, Glidewell O and Cooper RG (1980) Adverse effect of radiotherapy on adjuvant chemotherapy for carcinoma of the breast. *Surg Gynecol Obstet* **150**: 817–821

Hryniuk WM and Bush H (1984) The importance of dose intensity in chemotherapy of metastatic breast cancer. *J Clin Oncol* **2**: 1281–1287

Hryniuk WM and Levine MN (1986) Analysis of dose intensity for adjuvant chemotherapy trials in stage II breast cancer. *J Clin Oncol* **4**: 1162–1170

Hryniuk WM, Figueredo A and Goodyear M (1987) Applications of dose intensity to problems in chemotherapy of breast and colorectal cancer. *Semin Oncol* **14**: 3–11

Levine JF, Coleman NC, Cox RS, Ray GR, Rogoway WM, Martinez A, Stockdale FE (1984) The effect of post-operative and primary radiation therapy on delivered dose of adjuvant cyclophosphamide, methotrexate and 5-fluorouracil (CMF) chemotherapy in breast cancer. *Cancer* **53**: 237–241

Lieschke GJ and Burgess AW (1992) Drug therapy: granulocyte colony-stimulating factor and granulocyte-macrophage colony stimulating factor (Second of two parts). *N Engl J Med* **327**: 99–106

Lindeman GJ, Boyages J, Driessen C and Langlands AO (1992) Intravenous or oral adjuvant CMF for node-positive breast cancer. *Aust N Z J Surg* **62**: 556–562

Neidhart J, Mangalik A, Kohler W and Langlands AO (1989) Granulocyte colony-stimulating factor stimulates recovery of granulocytes in patients receiving dose-intensive chemotherapy without bone marrow transplantation. *J Clin Oncol* **7**: 1685–1692

Pronzato P, Miglietta L, Rubagotti A, Bertelli G, Queirolo P, Guenzi M, Sertoli MR, Vitale V, Rosso R (1993) Impact of irradiation of residual breast on adjuvant chemotherapy dose intensity. *Am J Clin Oncol* **16**: 58–60

Ribas A, Albanell J, Bellmunt J, Sole-Calvo LA, Bermejo B, Gallardo E, Vidal R, Vera R, Eres N, Carulla J, Baselga J (1996) Five-day course of granulocyte colony-stimulating factor in patients with prolonged neutropenia after adjuvant chemotherapy for breast cancer is a safe and cost-effective schedule to maintain dose-intensity. *J Clin Oncol* **14**: 1573–1580

Tannock IF, Boyd NF, De Boer G, Erlichman C, Fine S, Larocque G, Mayers C, Perrault D, Sutherland H (1988) A randomized trial of two dose levels of cyclophosphamide, methotrexate and fluorouracil chemotherapy for patients with metastatic breast cancer. *J Clin Oncol* **6**: 1377–1387

Tjan-Heijnen VC, Biesma B, Festen J, Splinter TA, Cox A, Wagener DJ, Postmus PE (1998) Enhanced myelotoxicity due to granulocyte colony-stimulating factor administration until 48 hours before the next chemotherapy course in patients with small-cell lung carcinoma. *J Clin Oncol* **16**: 2708–2714

WHO (1979) Handbook for reporting results of cancer treatment. WHO Offset Publication No. 48. Geneva, WHO

Wood WC, Budman DR, Korzun AH, Cooper MR, Younger J, Hart RD, Moore A, Ellerton JA, Norton L, Ferree CR, Colango Ballow A, Frei III E, Henderson IC (1994) Dose and dose intensity of adjuvant chemotherapy for stage II, node-positive breast carcinoma. *N Engl J Med* **330**: 1253–1259

*© 2000 Cancer Research Campaign*
